# Photoprotective Role of Neoxanthin in Plants and Algae

**DOI:** 10.3390/molecules25204617

**Published:** 2020-10-11

**Authors:** Chiara Giossi, Paulo Cartaxana, Sónia Cruz

**Affiliations:** CESAM–Centre for Environmental and Marine Studies, Department of Biology, University of Aveiro, Campus de Santiago, 3810-193 Aveiro, Portugal; chiara.giossi@ua.pt (C.G.); pcartaxana@ua.pt (P.C.)

**Keywords:** 9′-*cis*-neoxanthin, all-*trans*-neoxanthin, Bryopsidales, light harvesting complexes (LHC), photoprotection

## Abstract

Light is a paramount parameter driving photosynthesis. However, excessive irradiance leads to the formation of reactive oxygen species that cause cell damage and hamper the growth of photosynthetic organisms. Xanthophylls are key pigments involved in the photoprotective response of plants and algae to excessive light. Of particular relevance is the operation of xanthophyll cycles (XC) leading to the formation of de-epoxidized molecules with energy dissipating capacities. Neoxanthin, found in plants and algae in two different isomeric forms, is involved in the light stress response at different levels. This xanthophyll is not directly involved in XCs and the molecular mechanisms behind its photoprotective activity are yet to be fully resolved. This review comprehensively addresses the photoprotective role of 9′-*cis*-neoxanthin, the most abundant neoxanthin isomer, and one of the major xanthophyll components in plants’ photosystems. The light-dependent accumulation of all-*trans*-neoxanthin in photosynthetic cells was identified exclusively in algae of the order Bryopsidales (Chlorophyta), that lack a functional XC. A putative photoprotective model involving all-*trans*-neoxanthin is discussed.

## 1. Introduction: Xanthophylls’ Role in Photoprotection

Sunlight is essential to power the photochemical reactions of photosynthesis, but a surplus of excitation energy produces various radicals and reactive oxygen species (ROS) that can damage the photosynthetic apparatus, leading to a decline in photosynthetic activity, growth, and productivity (i.e., “photoinhibition”) [1,2]. Since light is not constant in nature, plants and algae have to cope with changing light environments that can potentially damage their photosynthetic activity [3,4]. Therefore, a quick and efficient response to light changes is essential for the survival and fitness of photosynthetic organisms in their variable natural environments.

Plants and algae have evolved a wide variety of photoprotective strategies that alleviate photoinhibition and are associated with avoiding light absorption and successfully consuming or dissipating the light energy absorbed by light-harvesting pigments (reviewed by [2,5]). In particular, non-photochemical quenching (NPQ) is a rapidly induced photoprotection mechanism (time-scale of seconds to a few minutes) that allows the dissipation of excessive excitation energy as heat (recently reviewed by [6,7]). NPQ consists of different components that contribute to the overall energy dissipation. The high-energy-state quenching (qE), known as the principal component of NPQ in plants, is related to the activity of a xanthophyll cycle (XC), a common photoprotection mechanism in plants and algae. The most common XC (known as the VAZ cycle), displayed by all land plants and several aquatic taxa, is based on the interconversion between the xanthophylls violaxanthin (V), antheraxanthin (A), and zeaxanthin (Z): under high light, V is converted into Z through the intermediate A (forward de-epoxidation reaction, catalyzed by the enzyme violaxanthin de-epoxidase—VDE), while under low light Z is converted back into V (reverse epoxidation reaction catalyzed by the enzyme zeaxanthin epoxidase—ZE) [8,9,10].

XCs are considered ubiquitous in the plant kingdom, but variations can be present, and different xanthophyll molecules can be involved: six different types of xanthophyll cycles have been described to date (for an extensive review on this topic see [11]). Despite their wide occurrence, the importance of XCs for photoprotection can vary between taxa: while their contribution to NPQ (in terms of qE) is known to be extremely relevant in higher plants, it was reported to be less significant in green algae [12]. For example, for the green microalgae *Chlamydomonas reinhardtii* it is known that the xanthophyll-mediated component qE is induced only after high light acclimation, while it is constitutive in higher plants [13]. Nonetheless, the role of xanthophylls in photoprotection is not limited to the XC: experimental evidence has shown that xanthophylls (e.g., zeaxanthin, lutein, neoxanthin) can be responsible for photoprotection in the form of general antioxidant activity, a mechanism non-related to NPQ [14,15,16]. Despite the widely recognized key role of xanthophylls in photoprotection, several questions are still open on the molecular mechanisms behind these processes.

Neoxanthin is one of the key photosynthetic xanthophylls in plants and algae. In this work, structure, biosynthesis, and distribution of the molecule are reviewed, and the biological role of 9′-*cis*-neoxanthin (the most abundant neoxanthin isomer in chloroplasts) is described. Also, we assess the putative photoprotective activity of all-*trans*-neoxanthin in siphonous green algae (Bryopsidales, Chlorophyta) that lack a functional xanthophyll cycle [17]. A photoprotective model that explains this activity and future research questions in this field are discussed.

## 2. Structure, Biosynthesis, and Distribution of Neoxanthin

### 2.1. Structure, Isoforms, and Chemical Properties

In the late 30s, Strain [18] first described the presence of a common, widely distributed xanthophyll in barley leaves, which he named neoxanthin. According to his experimental studies, this pigment (molecular formula: C_40_H_56_O_4_) was one of the three major carotenoids of plant leaves (together with lutein and violaxanthin) and had absorption properties close to that of violaxanthin (major peaks of absorption at 437 and 467 nm in ethanol). A tentative structure of the molecule was later proposed by Goldsmith and Krinsky [19], that identified the presence of one 5,6-epoxy and three hydroxyl groups and revised by Cholnoky et al. [20], that demonstrated the presence of an additional allenic group and confirmed the original molecular formula proposed by Strain. Finally, the structure of neoxanthin was confirmed through chemical synthesis [21] as the one we know today.

**Figure 1:**
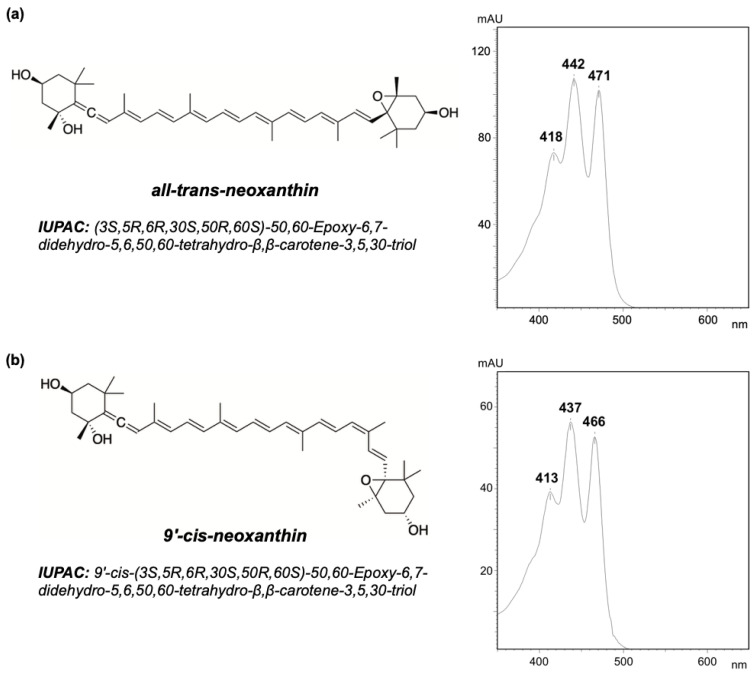
Molecular structure and representative absorption spectra of neoxanthin: (**a**) all-*trans*-neoxanthin and (**b**) 9′-*cis*-neoxanthin. Spectra were obtained from pigment extracts (95% methanol, 2% ammonium acetate) of a green macroalga (*Codium tomentosum*, Bryopsidales, Chlorophyta) analyzed with HPLC (Shimadzu, Kyoto, Japan). Separation was performed with the SUPELCOSIL C18 column (Sigma-Aldrich, St. Louis, MO, USA) for reverse phase chromatography, following a 35 min elution program as described by Mendes et al. [25].

Neoxanthin occurs mostly in two geometric isomers (Figure 1): all-*trans*-neoxanthin (also referred to as *trans*-neoxanthin) in which all double bonds have *trans* geometry, and 9′-*cis*-neoxanthin, in which the double bond at the 9′ position has *cis* geometry, while all the others have *trans* [22]. The two isoforms can be distinguished with chromatographical separation due to different absorption spectra: in ethanol, *trans*-neoxanthin presents major peaks of absorption at 418, 442, and 471 nm [21], while 9′-*cis*-neoxanthin at 413, 437, and 466 nm [23] (data relative to different extraction solvents are available in the literature [24]). Representative absorption spectra for both isomers (extracted in 95% methanol, 2% ammonium acetate) are shown in Figure 1.

Carotenoids are sensitive to acid, heat, light, and oxygen that can impair their biological activity. Neoxanthin is particularly labile to acids due to its epoxide nature: a bland acid treatment (e.g., 0.002% ethereal or chloroformic hydrogen chloride) leads to the production of a 5,8-furanoid oxide (called neochrome) as the main reaction product [20]. The same reaction (reorganization of the 5,6-epoxide group of neoxanthin to the 5,8-furanoxide group of neochrome) occurs via thermal degradation, but neoxanthin was reported to have higher thermal stability compared to other 5,6-epoxy xanthophylls (violaxanthin and antheraxanthin) [26].

### 2.2. Biosynthesis

The synthesis of neoxanthin in oxygenic phototrophs follows the general biosynthetic pathway of xanthophylls, divided into five steps [24]: (1) generation of active isoprene, building block of all isoprenoid molecules like carotenoids; (2) formation of the first carotenoid, phytoene, by stepwise condensation of eight isoprene units; (3) consecutive desaturation and isomerization reactions that lead to lycopene; (4) cyclization of the linear ends of lycopene yielding cyclic carotenes like α-carotene or β-carotene; (5) stepwise introduction of molecular oxygen creating a vast array of xanthophylls. The biosynthesis of xanthophylls can be divided into two main branches: those that derive from α-carotene (α-branch) and those that derive from β-carotene (β-branch).

Neoxanthin belongs to the β-branch of xanthophylls: first, β-carotene is converted to zeaxanthin due to the hydroxylation reaction at C-3 of both rings and catalyzed by the enzyme β-carotene 3,3′-hydroxylase [27]. Then, zeaxanthin is converted, via the intermediate antheraxanthin, into violaxanthin with an epoxidation of C-5 and C-6 of both rings catalyzed by the enzyme zeaxanthin epoxidase—ZE [10]. Finally, violaxanthin is converted into the allenic xanthophyll *trans*-neoxanthin, through a not completely understood reaction that is thought to involve *trans*-violaxanthin [28]. Furthermore, 9′-*cis*-neoxanthin might be subsequently produced via isomerization of *trans*-neoxanthin, but this reaction has still to be elucidated [29,30]. The existence of a reverse reaction from 9′-*cis* to *trans*-neoxanthin was to date not reported.

As for all carotenoids, neoxanthin synthesis takes place in the plastid, although controlled by nuclear-encoded genes [31]. The enzyme responsible for this process was termed neoxanthin synthase (NSY, also referred to as NXS). Genes encoding for a putative NSY were first reported in the potato and tomato, due to their ability to convert *trans*-violaxanthin into *trans*-neoxanthin in transient expression systems, and identified as paralogous of the lycopene cyclase (LCY), together with capsorubin synthase (CCS) from the bell pepper [29,32]. However, some studies yielded contrasting results: the NSY gene of the tomato was reported to be a fruit-specific LCY responsible for β-carotene production (named CYC-B), probably a bifunctional enzyme capable of converting both lycopene to β-carotene and violaxanthin to neoxanthin. Since neoxanthin is synthesized in mutants lacking CYC-B, the presence of a different NSY in the tomato was postulated [31,33]. Moreover, related orthologous genes were not found in other plants (*Arabidopsis thaliana*, *Oryza sativa*) and green algae (*Chlamydomonas reinhardtii*), leading to the conclusion that neoxanthin could be produced either via a secondary reaction operated by an LCY or via a completely different, unidentified NSY [31,34,35]. More recently, it was demonstrated that the *ABA4* gene of *A. thaliana,* related with abscisic acid (ABA) production, is involved in the accumulation of neoxanthin (particularly of the all-*trans*-isomer) [30]. In addition, a newly identified gene from the tomato (*Nxd1*) was reported to be necessary, but not solely sufficient, for neoxanthin synthesis in plants (the locus *nxd1*, whose function still needs to be completely elucidated, is conserved in all higher plants and some green algae) [36]. All these findings allow us to reason that the synthesis of neoxanthin from violaxanthin is a complex process, possibly governed by different genes. More comprehensive research on this topic is required to fully understand the dynamics between neoxanthin and related carotenoids within the photosystems of plants and algae.

Understanding neoxanthin biosynthesis is particularly relevant since it is related to the synthesis of ABA, an essential hormone in plants [28,37]. It has been demonstrated that ABA can be synthesized from different precursors, 9′-*cis*-neoxanthin and 9′-*cis*-violaxanthin (for an extended review see [38,39,40]), whose relative importance has long been debated. Additional studies demonstrated that the synthesis of ABA in vivo can depend on different precursors in plants [30,36,41]. Neoxanthin was also reported to act as an intermediate in the synthesis of peridinin and diadinoxanthin from zeaxanthin in a species of Dinophyceae (*Amphidinium carterae*) [42]. The involvement in the production of dinoxanthin (neoxanthin 3-acetate) was also postulated, but to date, the existence of this reaction was not proved [42,43].

### 2.3. Phylogenetic Distribution

With regard to phylogeny, a constitutive presence of neoxanthin was reported to be associated with that of chlorophyll *b*: it is absent in all algal taxa that lack chlorophyll *b* and in cyanobacteria (despite some genera contain chlorophyll *b*). Neoxanthin is known to be present in higher plants, ferns, mosses, green algae (Chlorophyta), and chlorophyll *b* bearing Dinophyceae, all containing both chlorophylls *a* and *b* [24,44]. As described above, neoxanthin appears to be involved in the production of characteristic xanthophylls of Dinophyceae, but in species that lack chlorophyll *b*, significant amounts of neoxanthin were never reported, leading to the conclusion that in these cases it is produced solely as a reaction intermediate [42]. It was shown that neoxanthin transfers light energy almost exclusively to chlorophyll *b* [45,46], therefore, possibly explaining the phylogenetic distribution of these two pigments. However, it is unclear whether their co-occurrence is functional or just coincidental.

The distribution of neoxanthin in oxygenic phototrophs also changes in relation to its geometrical isomerism: in plants, 9′-*cis*-neoxanthin is known to be the most abundant form in photosynthetic cells (leaves), while *trans*-neoxanthin was found only in non-photosynthetic organs (such as petals and fruits) [44,47]. Indeed, 9′-*cis*-neoxanthin was identified as one of the major xanthophyll components of plant photosystems, together with lutein and violaxanthin, and is the only one present in *cis* configuration [48,49,50,51]. A light-induced shift in the conformation of *cis*-neoxanthin (from 9′-*cis* to 9′,13-*dicis*, and 9′,13′-*dicis*) was observed in plants [26], with possible implications for photoprotection, as discussed in the following section.

The two geometrical isomers are also differentially distributed in algae: 9′-*cis*-neoxanthin is the dominant form in photosynthetic cells of almost all algal taxa, while *trans*-neoxanthin was reported to be the characteristic isomer of Mesostigmatophyceae, a peculiar class of ancient freshwater green flagellates with only two reported species under the genus *Mesostigma* [52,53]. The dominance of the 9′-*cis* form has been attributed to the fact that this isomer is one of the direct precursors of ABA (which is biologically active in *cis*- conformation [54]). Interestingly, a light-dependent conformational change of neoxanthin was observed in siphonous green algae of the order Bryopsidales: in response to high irradiance, *trans*-neoxanthin is accumulated in addition to the 9′-*cis* isomer [55,56,57]. The implications of this response for photoprotection are analyzed and discussed below.

## 3. Biological Role of 9′-*cis*-Neoxanthin

### 3.1. 9′-cis-Neoxanthin Binding Site

The xanthophyll, 9′-*cis*-neoxanthin, is considered one of the main photosynthetic xanthophylls in both green algae and higher plants and is present on the membrane in association with the thylakoid proteins: each light-harvesting complex of photosystem II (LHCII) generally binds four xanthophylls, namely two luteins, one violaxanthin, and one 9′-*cis*-neoxanthin [48,49,50,51]. These xanthophylls harvest light energy and transfer it to chlorophylls [58], but while lutein and violaxanthin transfer energy mainly to chlorophyll *a*, 9′-*cis*-neoxanthin transfers light energy almost exclusively to chlorophyll *b* [45,46]. The association of 9′-*cis*-neoxanthin with antenna proteins has been reported not only for the most known chlorophyll *a*/*b*-lutein LHCII (typical of land plants and most green algae), but also for other LHCII-types (chlorophyll *a*/*b*-siphonaxanthin [59], chlorophyll *a*/*b*-diadinoxanthin [60], and chlorophyll *a*/*b*-prasinoxanthin [61]). This suggests that while the composition of photosynthetic pigments can diversify through evolution, the role of 9′-*cis*-neoxanthin is conserved [44]. Interestingly, neoxanthin is the only photosynthetic carotenoid present in *cis* conformation, while all the other LHCII-bound carotenoids are found as all-*trans* [50,62].

The proteins of LHCII display a total of four binding sites for xanthophylls [50]: in plants, lutein and violaxanthin can compete for two binding sites (L1 and L2) that normally host lutein, located between helix B and A at the center of the molecule [48,63], and a weak peripheral binding site (V1) assigned to violaxanthin [51,64]; neoxanthin is bound to a highly selective site (N1) and contributes to but is not essential for the structural stability of the protein [49,50]. This binding site is located in a chlorophyll *b*-rich region around helix C of the LHCII protein and relies on a single hydrogen bond between the OH group at the tip of 9′-*cis*-neoxanthin and a lumenal-loop Tyr112 residue [50]. This bond is thermodynamically reversible, suggesting that LHCII-bound 9′-*cis*-neoxanthin could have a function in energy dissipation and/or represent a readily available source for ABA biosynthesis at elevated temperatures [65]. Experimental studies showed that the conformation of 9′-*cis*-neoxanthin is sensitive to changes in the surrounding molecular environment, including the thylakoid membranes [66] and the pigment-protein complex [67], with possible implications for energy harvest and transfer.

The structure of the N1 binding site appears to be conserved and highly specific for the 9′-*cis* stereoisomer of neoxanthin [62,68], with a relatively high binding affinity [65]. If neoxanthin is lacking (e.g., mutation), the N1 site can bind other *cis* carotenoids [15,69,70], indicating that the high specificity of this binding site depends on the *cis* geometry. Due to the protein folding around N1, the 9′-*cis*-neoxanthin molecule protrudes into the lipid phase of the membrane, with possible implications for the complex flexibility and interactions with other thylakoid proteins [51,62]. In particular, it has been shown that the protruding end ring of 9′-*cis*-neoxanthin has an important role in intermolecular interactions between major (LHCII) and minor (CP29, CP26) antenna proteins [71]. All these findings suggest that the properties of 9′-*cis*-neoxanthin binding can influence the plasticity of the protein-pigment complexes and the response to a dynamic light environment.

### 3.2. Role of 9′-cis-Neoxanthin against Photooxidative Stress: ^3^Chl* Quenching and ROS Scavenging

Besides the role of the light harvester and the structural contribution to the antenna complexes, 9′-*cis*-neoxanthin displays various physiological functions in LHCII: its connection with the complex’s proteins and the other photosynthetic pigments can influence how the energy is harvested, transferred, and dissipated through the photosystem, with interesting implications for photoprotection.

In general, when the antenna complexes are hit by an excess of light, excited singlet chlorophylls (^1^Chl*) can form excited chlorophyll triplets (^3^Chl*), leading to the production of singlet oxygen (^1^O_2_*), a potential source of oxidative stress [72]. Carotenoids in close interactions with chlorophylls can catalyze a chlorophyll-to-carotenoid triplet transfer (Dexter-type electron exchange [73,74]) that allows the quenching of ^3^Chl*, thus preventing the formation of ROS and the consequent photoinhibition [75]. In plants’ antennae, ^3^Chl* quenching is catalyzed by lutein [76,77] while 9′-*cis*-neoxanthin is not directly involved in this process [78]. However, experimental studies showed that the absence of 9′-*cis*-neoxanthin could reduce the efficiency of ^3^Chl* quenching, and this activity has been attributed to structural modifications of Lhc proteins. When the N1 site is empty, the interactions between chlorophylls and carotenoids become less tight, leaving some ^3^Chl* unprotected and opening direct access for oxygen to the end ring of lutein [79,80]. Therefore, the molecule of 9′-*cis*-neoxanthin occupying the N1 site acts as an oxygen barrier for the antenna core and is important for maintaining the close-distance interactions that allow ^3^Chl* quenching operated by lutein [79,80]. Accordingly, it was demonstrated that conformational changes of the LHCII-bound 9′-*cis*-neoxanthin can influence the interactions between this molecule, a neighboring chlorophyll *a* (Chl*a* 604), and the lutein at site L2, adjusting the triplet energy distribution and thus influencing ^3^Chl* quenching [81]. Structural studies also showed that conformational changes in the 9′-*cis*-neoxanthin-Tyr112 bond can strongly affect the site energy of Chl*a* 604 [82], confirming the importance of this molecule for balancing the energy transfer between photosynthetic pigments and for the photostability of the antenna complexes.

In addition to its indirect role in chlorophyll-to-carotenoid triplet transfer, 9′-*cis*-neoxanthin is directly involved in photoprotection as an antioxidant: antenna complexes lacking this molecule were reported to be more sensitive to photobleaching and showed a reduced capacity of ROS scavenging under excessive light [15,48]. It was first proposed that 9′-*cis*-neoxanthin could act as a direct scavenger of ^1^O_2_* diffusing from the chlorophyll *a* to the chlorophyll *b*-rich domain where the molecule is located [48]. On the other hand, mutants of *Arabidopsis thaliana* lacking 9′-*cis*-neoxanthin (aba4-1) showed the highest sensitivity to superoxide anions (O_2_^–^), indicating that the molecule might be particularly active against this type of ROS (produced by Mehler reaction [83]), despite signs of activity against ^1^O_2_* were also present [15]. In general, while its role in ^3^Chl* quenching seems to be more correlated with the occupancy of the N1 site rather than the molecule specificity [79], the ROS scavenging activity seems to be specifically dependent on the presence of 9′-*cis*-neoxanthin [15]. This is coherent with the theory of cooperative photoprotective action of xanthophylls: while some molecules can be interchanged without drastic alterations of the complex (e.g., replacement of 9′-*cis*-neoxanthin with 9′-*cis*-violaxanthin [15,69,70]), each one has specific functions that make it important for photoprotection and cells displaying the complete pool of xanthophylls are more resistant to light-induced stress [15,48].

### 3.3. Light-Induced Changes of 9′-cis-Neoxanthin and Photoprotection

In addition to its role in ^3^Chl* control and ROS scavenging, a light-induced isomerization of the LHCII-bound 9′-*cis*-neoxanthin has been correlated with photoprotection in plants [84]. In particular, illumination of isolated LHCII induces a reversible isomeric transition from the 9′-*cis* to the 9′-13- and the 9′-13′-*dicis* forms of neoxanthin; contextually a light-driven excitation quenching is observed [84]. This finding allowed to postulate that the isomeric transition of 9′-*cis*-neoxanthin to a *dicis* configuration could remove the steric hindrance and hydrogen bonding between protruding ends of neoxanthins from neighboring LHCII trimers, allowing excitonic and/or charge transfer interactions between the porphyrin rings of chlorophyll *b* molecules (termed Chl 605 [50] or Chl 14 [51]) [84]. This process would allow the quenching of singlet excitations in the antenna complexes, thus allowing a more efficient response to light stress.

Conformational changes of the 9′-*cis*-neoxanthin molecule have also been correlated with NPQ: it has been proposed that a light-induced twist in the Raman configuration of this pigment could induce allosteric modifications of LHCII, allowing the transition from the unquenched to the dissipative state of the antenna complex (a phenomenon that is still largely debated [85]), thus influencing the activation of NPQ [86,87]. A distortion of 9′-*cis*-neoxanthin has also been observed in the formation of LHCII oligomers [88] and in overcrowded grana thylakoids, where it has been correlated with unwanted energy dissipation and a reduction of energy transfer efficiency between antenna complexes [89]. In particular, it has been postulated that the conformational change induced by the 9′-*cis*-neoxanthin twist could alter the energy transfer equilibrium in the protein-pigment complex, increasing the energy transfer from excited chlorophylls to the molecule of lutein in site L1 and the consequent energy dissipation [86]. In support of this model, alterations of the neoxanthin/chlorophyll *b* domain were observed during the transition to the dissipative state of LHCII [90] and mutagenesis studies demonstrated that conformational changes of proteins in the lumenal loop (where N1 is located) can modulate the sensitivity of the complex to ∆pH, thus possibly influencing the formation of the NPQ cascade [91]. Therefore, despite 9′-*cis*-neoxanthin not directly participating in NPQ, changes in the protein conformation related to its binding site could be involved in this process.

Structural studies also suggested that conformational changes in the 9′-*cis*-neoxanthin-Tyr112 bond could alter the energy state of the neighboring Chla 604, and render it an alternative energy sink (“bottleneck”) in the lumenal layer of LHCII [82]. In principle, this chlorophyll could represent a quenching site, although this hypothesis is still debated [82,92]. Additional studies on the structural mobility of the lumenal loop of LHCII showed that the portion involving the N1 site is largely flexible, supporting the hypothesis of a local conformational change induced by the twist of 9′-*cis*-neoxanthin [93]. However, no significant differences were reported in complexes with and without neoxanthin [93], thus adding new questions to this already controversial topic.

The position of 9′-*cis*-neoxanthin in trimeric LHCII also influences the xanthophyll cycle in plants: it was shown that the protruding end of this pigment competitively interacts with the molecule of violaxanthin in the periphery of the complex (site V1) inhibiting its binding, thus accelerating the first reaction of the xanthophyll cycle (violaxanthin de-epoxidation) and promoting the formation of transient NPQ [94]. Despite all the unsolved questions, these findings confirm that rearrangements of 9′-*cis*-neoxanthin bound in site N1 could effectively contribute to the photostability of LHCII trough different molecular interactions regulating the excitation energy that is transferred and/or quenched in the photosystems.

## 4. All-*trans*-Neoxanthin in Bryopsidales (Chlorophyta): A New Putative Photoprotective Role

### 4.1. Peculiarities of Bryopsidales Light Harvesting Complexes and Absence of a Xanthophyll Cycle

In green algae of the order Bryopsidales, the chlorophyll *a*/*b* light-harvesting complexes (LHC) include a unique set of light-harvesting carotenoids: siphonaxanthin and its ester siphonaxanthin-dodecenoate (also referred to as siphonein) are found instead of lutein [95,96,97,98], normally present in higher plants [62]. For this reason, the core of Bryopsidales’ LHC was termed siphonaxanthin-chlorophyll *a*/*b* protein (SCP) [55]. Since this structure is found in ancient species, and siphonaxanthin is supposed to be an ancestor of lutein, SCPs have been suggested to be ancient LHC evolved in deep water green algae, therefore, representing an evolutionary relic of some Chlorophyta [96,99]. This particular organization, coupled with an increment of the chlorophyll *b*:*a* ratio, is thought to be responsible for enhancing the absorption spectra in the blue-green region, allowing the harvesting of light wavelengths that represent the predominant light in deep waters and shaded subtidal marine habitats, where these organisms live [95,96,97,98].

The pigment pool of SCPs is composed mainly of chlorophylls and siphonaxanthin, with the addition of accessory carotenoids, which can vary between species and between the two photosystems. For example, isolated SCPs from PSII of *Bryopsis* spp. were reported to include six molecules of chlorophyll *a*, eight of chlorophyll *b* and five xanthophylls (three molecules of siphonaxanthin, one siphonein, and only one of the accessory xanthophyll neoxanthin) per polypeptide [59,98], while in PSI the presence of different accessory carotenoids was reported (namely neoxanthin, violaxanthin, ε-carotene, and α-carotene) [100].

In addition to the peculiar organization of its antenna complexes, some studies have also suggested that Bryopsidales lack a functional xanthophyll cycle (XC), a common photoprotective mechanism in plants and algae, that allows the dissipation of excessive excitation energy as heat [8,9,10,11]. Experimental evidence first suggested that the VAZ cycle seemed to be inactive or functionally absent in different Bryopsidales (*Chlorodesmis* spp., *Caulerpa* spp., *Codium* spp.) [101,102,103]. Following these first results, it was later demonstrated that, although XC pigments are present, NPQ formation is independent of an XC in several Bryopsidales species, showing that this feature seems to be widespread in the order [17,104]. These findings raised different questions regarding how these species compensate for the absence of such a relevant NPQ component and which mechanisms are involved in photoprotection in this monophyletic branch of algae.

### 4.2. trans-Neoxanthin Is Accumulated in Bryopsidales under High Light

Given the unique composition of Bryopsidales’ LHC, attention has been addressed to the changes in their pigment composition under different light regimes. In particular, it was first reported that *Codium intricatum* displayed a light-dependent conformational change of the pigment neoxanthin: under high light acclimation, in addition to the normal pool of 9′-*cis*-neoxanthin (present as a major photosystems’ component in plants and algae [49,50,51]), a significant amount of all-*trans*-neoxanthin was accumulated, contextually with a relative increase of violaxanthin [55]. To date, a similar shift in the composition of the neoxanthin pool was not reported in any other photosynthetic taxon. These findings were later confirmed by separated studies, involving different Bryopsidales species (*Codium tomentosum* and *Bryopsis plumosa*) [56,57].

**Figure 2:**
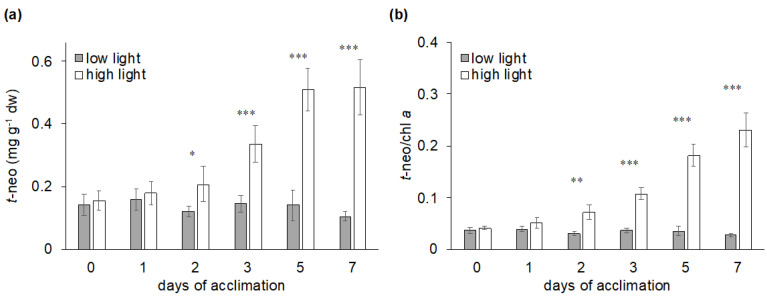
All-*trans*-neoxanthin (*t*-neo) content of low light (control, 20 μmol photons m^−2^ s^−1^) and high light (1000 μmol photons m^−2^ s^−1^) acclimated *Bryopsis plumosa,* during one week of exposure to the different light conditions, under long day photoperiod (16:8 h) at 17 °C. (**a**) Total amount of all*-trans*-neoxanthin per dry weight (mean ± SD; *n* = 5); (**b**) all*-trans*-neoxanthin/chlorophyll *a* ratio (mean ± SD; *n* = 5). Pigment extracts (95% methanol, 2% ammonium acetate) were analyzed with HPLC (Shimadzu, Japan), separation performed with SUPELCOSIL C18 column (Sigma-Aldrich, St. Louis, MO, USA) for reverse phase chromatography, following a 35 min elution programme as described by Mendes et al. [25]. Asterisks indicate significant differences between low light and high light acclimation conditions (*t*-tests; * *p* < 0.05, ** *p* < 0.01, *** *p* < 0.001).

Recently [57], it was also shown that all-*trans*-neoxanthin is not only constitutively produced under high light, but that its accumulation is positively correlated with the duration of high light exposure (Figure 2), suggesting that the intensity of the response might depend on the extent of the all-*trans*-neoxanthin pool. Moreover, this study revealed that the pigment accumulation response could be faster than previously thought: under exposure to high light (1000 μmol photons m^−2^ s^−1^) significant amounts of all-*trans*-neoxanthin are accumulated within two days (Figure 2), while under lower irradiance conditions (200 to 500 μmol photons m^−2^ s^−1^) the pigment accumulation was reported after one to two weeks [55,56]. These results suggest that the algae, in response to different light conditions, could efficiently modulate the light-induced all-*trans*-neoxanthin pool. However, it is conceivable that species-specific and environmental features would determine variability in this response, and further studies involving multiple Bryopsidales species are needed to corroborate these findings.

### 4.3. The Photoprotective Model

**Figure 3:**
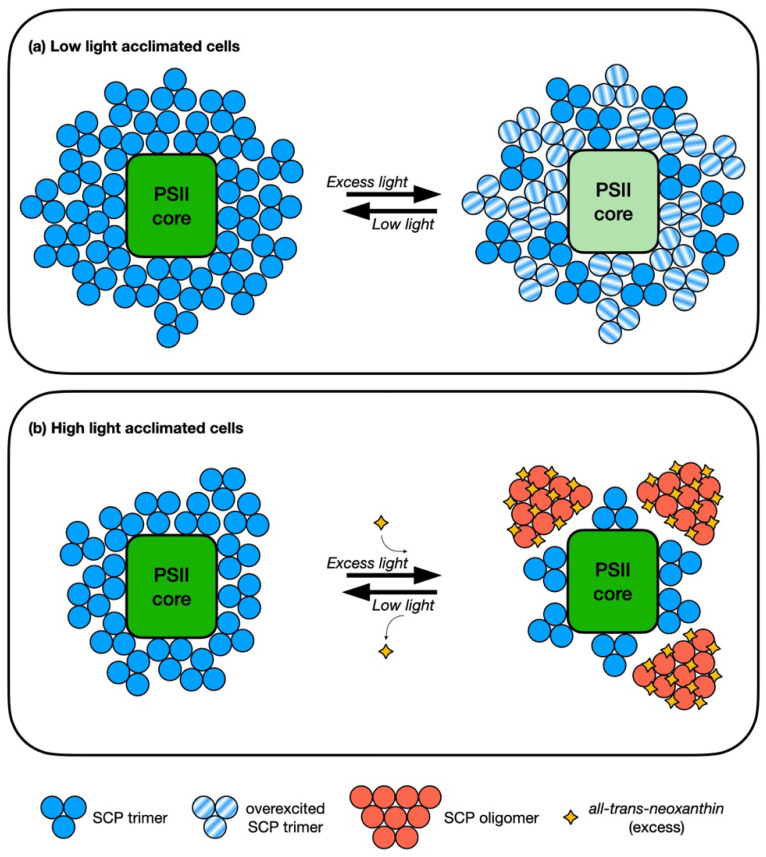
Putative photoprotective activity of *trans*-neoxanthin in Bryopsidales that lack a functional XC [17], according to the model proposed by Uragami et al. [55]. (**a**) In low light acclimation conditions, LHCII complexes form trimers directly connected with the PSII core. In the LCHII complexes, the photosynthetic pigments bound to SCPs (chlorophyll *b*, siphonaxanthin, and siphonein) harvest light energy and transfer it to chlorophyll *a*. Subsequently, the excitation energy is transferred from the SCP-bound chlorophyll *a* in LHCII to the reaction centers of PSII. However, when exposed to an excess of light the SCPs can accumulate a surplus of excitation energy, possibly leading to photoinhibition and photodamage. (**b**) Under acclimation to high irradiance, an excessive amount of *trans*-neoxanthin is accumulated constitutively. When the high light acclimated cell is exposed to an excess of light, these pigments bind to the surface of SCPs and promote the oligomerization of LHCII complexes, controlling the energy transferred to the PSII reaction center and allowing the quenching of the surplus of excitation energy.

Uragami and co-workers [55] were the first to postulate a role for the observed accumulation of the *trans*-isomer of neoxanthin under high irradiance in Bryopsidales. In their work, it was shown that the assembly of PSII SCPs of *Codium intricatum* changed under different light intensities: the complex was trimeric under low light acclimation, and oligomeric under high light. It was also shown that the interaction between the newly accumulated *trans*-neoxanthin (and violaxanthin) and SCPs were weak. These findings allowed for the postulation that the excess of *trans*-neoxanthin (and violaxanthin), synthesized under high light conditions, would bind to the surface of SCP and promote the oligomerization of the complex. This process could allow the control of the amount of energy transferred from SCP to PSII by adjusting the distance between the energy donor and the energy accepter, in order to quench the excess amount of excitation energy. The weak bond between the pigment and the protein would allow a fast and flexible response. The proposed model is represented in Figure 3.

The formation of an energy-quenching site through the aggregation of the LHC complexes was already reported in other photosynthetic organisms. In diatoms, one of the two specific quenching sites that are formed under high light is located in the oligomeric LHCII complexes (formed by FCPs—fucoxanthin-chlorophyll-proteins), which are functionally detached from the photosystems as a response to high light [105]. This quenching site would allow the fast dissipation of excess light through inter-complex pigment–pigment interactions, induced by conformational changes within the oligomerized antenna and acting independently from the xanthophyll cycle [6,105]. The quenching could either be determined by chlorophyll–chlorophyll interactions [106] or by chlorophyll-fucoxanthin interaction, attributing a new photoprotective role to the light-harvesting pigment fucoxanthin [6,107].

A corresponding mechanism was also reported in plants: it was postulated that one quenching site, located in functionally detached LHCII oligomers and strictly dependent on the PsbS protein, could generate energy dissipation independently from the xanthophyll cycle [108]. Indeed, conformational modifications in the LHCII oligomer would lead to the formation of dissipative pigment interactions: the newly formed chlorophyll–chlorophyll or chlorophyll–carotenoid interactions would determine energy quenching by charge transfer (between chlorophylls) or energy transfer (from the chlorophyll to the carotenoid) d. Interestingly, the transition into the dissipative oligomeric LHCII state has been shown to induce a distortion in the three-dimensional configuration of the associated 9′-*cis*-neoxanthin [88].

It is possible that a quenching site, functionally similar to that of detached FCP (in diatoms) and LHCII (in plants) oligomers, is present within the SCP oligomers of Bryopsidales, formed in the presence of all*-trans*-neoxanthin. Since a xanthophyll cycle was shown to be functionally absent in several Bryopsidales species [17], the existence of this mechanism would represent a safety valve for excess energy dissipation under extreme light conditions. However, there is currently no direct evidence of such quenching activity within the SCP oligomers and of the role of all*-trans*-neoxanthin (that could just act as a promoter of the oligomerization or have a direct role in the dissipative pigment-pigment interactions). It has to be noted that PSII could benefit from sufficient photoprotection thanks to the sole detachment of LHCX complexes (i.e., antenna size reduction), without the intervention of a hypothetical quenching site. Further characterization of protein–pigment interactions and possible NPQ development within SCP trimers and oligomers, under different light exposure and acclimation conditions, are required to assess this issue.

Another open question concerns the role of violaxanthin in this photoprotective model. This pigment was shown to be accumulated under high light acclimation together with all-*trans*-neoxanthin [55,56,57] and both these 5,6-epoxy xanthophylls can be bound to SCP (despite the association of violaxanthin in isolated LHC complexes being reported less frequently compared to that of neoxanthin) [59,98,100]. Nonetheless, violaxanthin is known as the direct precursor of neoxanthin biosynthesis d. Therefore, it is unclear whether violaxanthin is involved in the photoprotective model described above, contributing to the promotion of SCPs oligomerization and/or to the related energy dissipation, or if it is accumulated only as a biosynthetic precursor to sustain the production of all-*trans*-neoxanthin.

## 5. Conclusions and Future Research Perspectives

Strong evidence has been found for a photoprotective role of the xanthophyll pigment neoxanthin in plants and algae. One of the main xanthophyll components in the photosystem of all chloroplasts [44], 9′-*cis*-neoxanthin, is thought to be involved in different mechanisms of excess energy dissipation, despite its role can appear more cryptic compared to that of other photosynthetic xanthophylls. While progress has been made in this field, several questions still remain unsolved, in particular regarding the molecular processes at the base of the light-induced isomeric transitions [84] and conformational changes [86] of the LHCII-bound 9′-*cis*-neoxanthin, and their involvement in photoprotection and in the regulation of NPQ.

In Bryopsidales, a group of algae that lack the energy dissipating xanthophyll cycle, accumulation of all-*trans*-neoxanthin during high light stress indicates a photoprotective role of this isomer. In the photoprotective model discussed [55], all-*trans*-neoxanthin promotes the oligomerization of the light-harvesting complexes, thus influencing the amount of energy that is transferred to PSII. However, the validity of the model requires further experimental evidence and a direct correlation between the accumulation of all-*trans*-neoxanthin and photoprotection has still to be assessed. This could be achieved by investigating if and how the presence of all-*trans*-neoxanthin reduces photodamage to protein D1 (main target of photoinhibition in PSII [109]) and/or prevents the formation of ROS. All-*trans*-neoxanthin might actually have multiple functions in Bryopsidales’ cells exposed to high light, possibly participating both directly (quenching/regulation of energy transfer at the level of SCP oligomers) and indirectly (e.g., in the form of antioxidant activity) in the dissipation of excess light energy and in the light stress response.
